# Evaluation of the quotient of the venoarterial carbon dioxide gradient and the arteriovenous oxygen content difference as a transfusion trigger parameter in hemodynamically stable patients with significant anemia

**DOI:** 10.1186/cc14411

**Published:** 2015-03-16

**Authors:** A Taha, A Shafie, M Mostafa, N Syed, H Hon, R Marktanner

**Affiliations:** 1 Sheikh Khalifa Medical City, Abu Dhabi, United Arab Emirates

## Introduction

Hemoglobin as the main trigger parameter for blood transfusion usually gives diminutive information about oxygen delivery and consumption. Although central venous oxygen saturation (ScvO_2_) is an alternative parameter, its changes are unable to detect regional hypoxia. Our aim was to evaluate the quotient of the central venous-toarterial carbon dioxide gradient (δPCO_2_) and the arteriovenous oxygen content difference (Ca-cvO_2_) as a valid transfusion trigger parameter in hemodynamically stable anemic patients to reduce the amount of potentially counterproductive erythrocyte transfusions [1].

## Methods

Forty-five postoperative patients admitted to our cardiac ICU were enrolled between January 2013 and September 2014. Three groups were defined according to the trend of blood loss over the surgical drains in the first 24 postoperative hours. Mild blood loss was defined as 500 to 1,000 ml/24 hours, moderate (1,000 to 1,500/24 hours) and severe (>1,500 ml/24 hours). In addition to the δPCO_2_ the following parameters were monitored: CI, CO, SVR, serum lactate, ScvO_2_ and hemoglobin. Ca-cvO_2_ was calculated and the δPCO_2_/ Ca-cvO_2_ quotient was assessed for a total of 400 paired blood samples. All enrolled patients were hemodynamically stable. A retrospective analysis of this data was performed.

## Results

δPCO_2_/Ca-cvO_2_ showed significant correlation with the moderate and severe blood loss groups (*P *< 0.01), while no significant correlation was detected in the mild blood loss group. The abnormality of the δPCO_2_/Ca-cvO_2_ was easy detectable and reflected intracapillary hemoglobin capacity decline and significantly improved after erythrocyte transfusions (*P *< 0.005).

## Conclusion

Blood transfusions carry risks of adverse effects and should be carried out responsibly. Our findings suggest an additive and easy detectable transfusion trigger parameter (δPCO_2_/Ca-cvO_2_) providing physiological information on anemia-related altered oxygen extraction conditions and hence the indication for erythrocyte transfusions. However, additional studies are warranted to confirm these findings.
